# Randomised Controlled Trial to determine the appropriate time to initiate peritoneal dialysis after insertion of catheter to minimise complications (Timely PD study)

**DOI:** 10.1186/1471-2369-11-11

**Published:** 2010-06-22

**Authors:** Dwarakanathan Ranganathan, Richard Baer, Robert G Fassett, Nicola Williams, Thin Han, Melanie Watson, Helen Healy

**Affiliations:** 1 Renal Medicine, Royal Brisbane and Women's Hospital, Brisbane, Queensland, 4029, Australia; 2 Renal Medicine, Cairns Base Hospital, Cairns, Queensland, 4870, Australia; 3 School of Medicine, The University of Queensland, Brisbane, Queensland, 4027 Australia; 4 School of Human Movement Studies, The University of Queensland, Brisbane, Queensland, 4027, Australia; 5 Renal Medicine, Rockhampton Base Hospital, Rockhampton, Queensland, 4700, Australia; 6 Statistical Analysis Unit, Health Statistics Centre, Queensland Health, Queensland, 4000, Australia

## Abstract

**Background:**

The most appropriate time to initiate dialysis after surgical insertion of Tenckhoff catheters is not clear in the literature. There is the possibility of peritoneal dialysis (PD) complications such as leakage and infection if dialysis is started too soon after insertion. However, much morbidity and expense could be saved by reducing dependency on haemodialysis (HD) by earlier initiation of PD post catheter insertion. Previous studies are observational and mostly compare immediate with delayed use. The primary objective is to determine the safest and shortest time interval between surgical placement of a Tenckhoff catheter and starting PD.

**Methods/Design:**

This is a randomised controlled trial of patients who will start PD after insertion of Tenckhoff catheter at Royal Brisbane and Women's Hospital (RBWH) or Rockhampton Base Hospital (RBH) who meet the inclusion criteria. Patients will be stratified by site and diabetic status. The patients will be randomised to one of three treatment groups. Group 1 will start PD one week after Tenckhoff catheter insertion, group 2 at two weeks and group 3 at four weeks. Nurses and physicians will be blinded to the randomised allocation. The primary end point is the complication rate (leaks and infection) after initiation of PD.

**Discussion:**

The study will determine the most appropriate time to initiate PD after placement of a Tenckhoff catheter.

**Trial Registration:**

ACTRN12610000076077

## Background

Peritoneal dialysis (PD) catheters are inserted surgically or laparoscopically into the peritoneal cavity for the initiation of PD. The best time to use a PD catheter after its insertion is not clear from the literature. Complications such as dialysate fluid leaks and infection at the exit site or peritonitis can occur if PD is initiated too soon after insertion of the catheter [1]. Bridging hemodialysis (HD) may be required if PD is delayed [2]. Morbidity and expense could be saved by earlier initiation of PD post Tenckhoff catheter insertion that would limit the requirement for bridging HD. Current practice varies widely. Studies reported in this area have been observational and mostly compare immediate with delayed use[3,4]. Some studies support delayed use but do not test different delay intervals and can not tell us the optimal time to initiate PD [5-7]. Recent studies report a very low leak incidence after short break in period, attributed to tight catheter securing [8,9]. However, there is a lack of control groups in all these studies. A single centre retrospective and subsequent prospective audit found less complications when dialysis was initiated two weeks after Tenckhoff catheter insertion compared with immediate use [10].

Delay in starting PD results in the need for bridging HD in patients who require immediate dailysis. Bridging HD usually requires temporary HD catheters, with attendant risk of complications [11] and is also more expensive than PD [12].

Guideline bodies, such as Caring for Australasians with Renal Impairemnt (CARI) recommend, based on Level III and IV evidence, starting PD two weeks post Tenckhoff catheter insertion and advocate randomised controlled trials and further research [13]. International guidelines (European Dialysis and Transplant Association-European Renal Association and International Society for Peritoneal Dialysis, 1998 [14]) also suggest a two week delay and state that there is insufficient evidence to formulate a "guideline".

Comparing lag periods of one, two and four weeks after insertion of Tenckhoff catheters will enable us to determine how much effect the duration of the delay has, if any, on PD complications while determining any advantage that earlier start may have in terms of limiting HD during the bridging period.

### Aim

The primary purpose of this study is to find out the optimum time required to initiate PD after Tenckhoff catheter insertion. This will be done by measuring the incidence of PD complications in a period post PD commencement as well as the HD associated problems during the same period if bridging HD is performed.

## Methods/Design

### Study design

This is a randomised controlled trial to determine the optimal time to initiate PD after insertion of a Tenckhoff catheter.

Patient population: All patients undergoing PD catheter insertion at Royal Brisbane and Women's Hospital(RBWH) and Rockhampton Base Hospital (RBH), with the intention to use the catheter as soon as possible, will be invited to participate in the study.

Intervention: The interventions will comprise three groups. The first group will start PD at one week, the second group at two weeks and the third group at four weeks after the insertion of a Tenckhoff catheter.

### Setting

The Department of Renal Medicine, RBWH and RBH service populations of approximately 1.2 million and 200,000 respectively. This study is conducted by the 'Home Independent dialysis and Transition Program' (HITS) of the Department of Renal Medicine, RBWH and the Peritoneal Dialysis Service of RBH.

### Identification of eligible patients

Patients who satisfy the inclusion and exclusion criteria will be recruited from pre-dialysis clinics, PD clinics, catheter insertion preadmission clinics, catheter insertion operating lists and the HD population. Written informed consent will be obtained from all participants.

### Inclusion criteria

Male and female participants over 18 years of age, who will be receiving Continuous Ambulatory Peritoneal Dialysis (CAPD) or Automated Peritoneal Dialysis (APD) within four weeks of insertion of a PD catheter, will be eligible for this study.

### Exclusion criteria

Participants will be excluded if there is a history of psychological illness or condition which interferes with their ability to understand or comply with the requirements of the study or if they had an acute infectious episode in the last month before enrolment.

### Participants

Participants will be stratified by site and diabetic status. The patients will then be block randomised in equal proportions to the three treatment groups using randomly varying blocks of size three and six. The randomisation sequence was generated using Stata 11 software [15]. Group 1 will start dialysis one week after Tenckhoff catheter insertion, group 2 at two weeks and group 3 at four weeks. A research nurse independent of study nurses and physicians will undertake randomisation process.

Patients will initiate dialysis in equivalent fashion apart from the study variable of delay period before initiation. The patients will be trained by the PD nurses in their respective centres. The PD exit site and tunnel will be examined according to the usual clinical practice at each site to detect any complications. This will not interfere with routine dressing changes or reviews currently in practice.

### Outcome measures

#### Primary

Peritoneal fluid leaks (over the four weeks following start up of PD) and/or PD related infection - either exit site and/or tunnel and/or peritonitis - over the four weeks following start up of PD (Table 1).

**Table 1:** Explanation of definitions to be used as part of this study

Terms	Definitions
Dialysate leak [17]	Exit-Site leak: Appearance of any moisture around the PD catheter that can be identified as dialysate (leak that contains a high glucose concentration, documented by a positive glucose dipstick of the leaking fluid)
	Dialysate leaks include not only those occurring around catheter but also dialysate loss from the peritoneal cavity, except that via the lumen of the PD catheter. If the diagnosis is uncertain Tenckhoffogram (intraperitoneal infusion of contrast material through the catheter with computed tomography) performed to confirm the anatomical leak[18]

Peritonitis [19]	Presence of two clinical signs and symptoms:
	• abdominal pain, nausea, vomiting, diarrhoea, fever and cloudy dialysate
	• Peritoneal dialysate WCC > 100/mm^3 ^with 50% neutrophils
	• Demonstration of bacteria on gram stain or culture

Exit site Infection (ESI)	Presence of purulent discharge or two of three of the following: erythema of >13 mm, induration and tenderness. Exit- site swab will be obtained in all suspected cases of ESI.

Tunnel Infection	Presence of two of three of the following: induration, tenderness, and radiographic evidence of a collection along the PD catheter tunnel.

#### Secondary

Composite of all PD catheter related complications and HD requirements and associated complications. PD catheter related complications include fluid leaks, infection, cuff erosion, cuff extrusion, failure to establish PD successfully (may be due to blockage, fibrin plug or outflow obstruction), delayed wound healing, haematoma, catheter revision or replacement, conversion to HD, HD related events, allergic reactions, hospitalisation for any reason with cause recorded as related to either surgery or start up of dialysis and death during the study period.

Outcomes will be recorded for eight weeks following the insertion of the Tenckhoff catheter. However, primary outcomes will relate only to those events occurring within four weeks after start up of dialysis as these will best inform the question of optimal delay time.

The HD related events include the number of HD treatments required during the study period, the use of temporary HD vascular access (non-tunnelled and tunnelled central venous catheters) and a composite of HD vascular access complications - infection (exit site or bacteraemia or septicaemia), blockage requiring intervention eg urokinase, replacement, repositioning and/or removal

### Data collection

Data will be obtained by history from the participant or extracted from the clinical notes. These include:

1. Participant information - age, gender, weight, height, allergies, cause of kidney disease, co-morbidities

2. Date of PD catheter insertion and start of this period of peritoneal dialysis

3. Type of PD catheter and the technique by which PD catheter was inserted

4. Previous dialysis history

5. PD related complications

6. HD related events

7. Laboratory investigations - full blood count, electrolytes, coagulation profile, liver function test and C-reactive protein at admission to hospital as part of usual care.

8. Microbiology results - Gram stain, culture and antimicrobial sensitivities of dialysate fluid at admission to hospital if there is clinical suspicion of peritonitis

9. Antibiotics and other drugs prescribed concomitantly

10. Length of stay in hospital.

### Statistical considerations

The proposed sample size is 315 patients, 105 per group. A prospective study conducted in a single centre (RBWH) showed a complication rate of 38% with immediate use and 2.56% with a two week delay period [9]. Unpublished data of complication rates with four weeks delay period were quoted as 1-2 in 30 cases. To obtain 80% power with an overall 1-sided significance level of 0.05, approximately 105 patients are required per group to establish non-inferiority, defined as a difference in complication rates less than 10% between two groups. It was assumed that the expected complication rate was 3% as estimated from the preliminary data, and the significance level was adjusted for multiple comparisons. Sample size calculations were performed using ACCorD (Analysis of Censored and Correlated Data, Version 1.6.9, 2010).

### Data analysis

The outcome variables, both primary and secondary, are binary. Hence the outcomes will be analysed using logistic regression to compare the three arms of time to dialysis, adjusting for diabetes or not. Outcomes will be analysed using the statistical program SPSS.

### Ethical Considerations

The Human Research and Ethics Committee of the RBWH (HREC/2007/171) and Human Research Ethics Committee of the Rockhampton Base Hospital (HREC09/QCQ29) approved this study

### Withdrawal from Study

Participants may withdraw from the study anytime without prejudice, as documented and explained at the time of consent.

## Discussion

This study targets patients with end stage kidney disease who choose PD for maintenance therapy. Current practice at some centres and at the RBWH [10] is to wait four weeks after a Tenckhoff catheter is placed before starting PD. A few centres start PD immediately after insertion [16] or after a week. Several Australian centres wait for two weeks and the CARI guidelines suggest a two-week delay. There is no consensus in the literature on optimal time interval between peritoneal catheter insertion and start up of dialysis. This is a randomised study to compare the complication rates in one, two, and four weeks delay in initiating PD from the time of insertion of PD catheter. Safety will be measured by assessing adverse events as a consequence of starting PD. The hypothesis is that there is no difference in complication rate between one, two and four weeks delay. If non-inferiority is demonstrated between one, two and four weeks delay in this trial then we could justifiably decrease the duration of delay significantly. The benefit to future patients would be fewer HD episodes, shorter duration of HD catheter placement, fewer HD catheter infections and fewer HD catheter replacement requirements. Results showing more complications in the one or two week delay groups will influence the current international guidelines, based on an appropriate risk/benefit analysis.

## List of abbreviations

IP: Intraperitoneal; ISPD: International Society for Peritoneal Dialysis; PD: Peritoneal dialysis; CARI: Caring for Australians with Renal impairment; ESRD: End Stage Renal disease; HD: Hemodialysis

## Competing interests

The authors declare that they have no competing interests.

## Authors' contributions

RB, HH, DR designed the study and wrote the protocol. RGF provided advice and input and will be involved with the study. TH will lead the Rockhampton centre. NW will be involved with the recruitment and data collection component of the study. MW provided the sample size calculation, reviewed study design, randomisation process and will perform the data analysis. All authors read and approved the final manuscript.

## Pre-publication history

The pre-publication history for this paper can be accessed here:

http://www.biomedcentral.com/1471-2369/11/11/prepub
